# Vagal Stimulation Ameliorates Non-Alcoholic Fatty Liver Disease in Rats

**DOI:** 10.3390/biomedicines11123255

**Published:** 2023-12-08

**Authors:** Hany A. Elkattawy, Samar Mortada Mahmoud, Ahmed El-Sayed Hassan, Ahmed Behiry, Hasnaa Ali Ebrahim, Ateya Megahed Ibrahim, Donia Elsaid Fathi Zaghamir, Mohamed El-Sherbiny, Sherein F. El-Sayed

**Affiliations:** 1 Department of Basic Medical Sciences, College of Medicine, AlMaarefa University, Riyadh 11579, Saudi Arabia; hmohammed@um.edu.sa; 2 Medical Physiology Department, College of Medicine, Zagazig University, Zagazig P.O. Box 44519, Egypt; a.hassan@sr.edu.sa (A.E.-S.H.); sfhussein@medicine.zu.edu.eg (S.F.E.-S.); 3 Department of Human Anatomy and Embryology, College of Medicine, Zagazig University, Zagazig P.O. Box 44519, Egypt; smmurtada@medicine.zu.edu.eg; 4 Department of Basic Medical Sciences, College of Medicine, Sulaiman Al-Rajhi University, Bukayriah 51941, Saudi Arabia; 5 Department of Tropical Medicine and Endemic Diseases, College of Medicine, Zagazig University, Zagazig P.O. Box 44519, Egypt; asbehery@medicine.zu.edu.eg; 6 Department of Basic Medical Sciences, College of Medicine, Princess Nourah bint Abdulrahman University, Riyadh 11671, Saudi Arabia; haebrahim@pnu.edu.sa; 7 Department of Nursing, College of Applied Medical Sciences, Prince Sattam bin Abdulaziz University, Al-Kharj 11942, Saudi Arabia; a.eleglany@psau.edu.sa (A.M.I.); d.zaghamir@psau.edu.sa (D.E.F.Z.); 8 Department of Family and Community Health Nursing, Faculty of Nursing, Port Said University, Port Said P.O. Box 42511, Egypt; 9 Department of Pediatric Nursing, Faculty of Nursing, Port Said University, Port Said P.O. Box 42511, Egypt

**Keywords:** high-fat diet, obesity, non-alcoholic fatty liver, vagus nerve

## Abstract

Background: The harmful consequences of non-alcoholic fatty liver disease (NAFLD) are posing an increasing threat to public health as the incidence of diabetes and obesity increases globally. A non-invasive treatment with a range of autonomic and metabolic benefits is transcutaneous vagus nerve stimulation (tVNS). Aim of the study: To investigate the possible preventive impacts of VNS against adult rats’ NAFLD caused by a high-fat diet (HFD) and to clarify the underlying mechanisms. Methods: A total of thirty-two adult male rats were split into two groups: the HFD-induced NAFLD group (*n* = 24) and the control normal group (*n* = 8). The obesogenic diet was maintained for 12 weeks to induce hepatic steatosis. The HFD-induced NAFLD group (*n* = 24) was separated into three groups: the group without treatment (*n* = 8), the group with sham stimulation (*n* = 8), and the group with VNS treatment (*n* = 8). VNS was delivered for 30 min per day for 6 weeks after the establishment of NAFLD using a digital TENS device. The subsequent assessments included hepatic triglyceride, cholesterol content, serum lipid profile, and liver function testing. In this context, inflammatory biomarkers (TNF-α, IL-6) and hepatic oxidative stress (MDA, SOD, and GPx) were also assessed. To clarify the possible mechanisms behind the protective benefits of VNS, additional histological inspection and immunohistochemistry analysis of TNF-α and Caspase-3 were performed. Results: In the NAFLD-affected obese rats, VNS markedly decreased the rats’ body mass index (BMI) and abdominal circumference (AC). Liver function markers (albumin, ALT, and AST) and the serum lipid profile—which included a notable decrease in the amounts of hepatic triglycerides and cholesterol—were both markedly improved. Additionally, oxidative stress and inflammatory indicators showed a considerable decline with VNS. Notably, the liver tissues examined by histopathologists revealed that there is evidence of the protective impact of VNS on the oxidative and inflammatory states linked to HFD-induced NAFLD while maintaining the architectural and functional condition of the liver. Conclusions: Our findings suggest that VNS may represent a promising therapeutic candidate for managing NAFLD induced by obesity. It can be considered to be an effective adjuvant physiological intervention for the obese population with NAFLD to spare the liver against obesity-induced deleterious injury.

## 1. Introduction

In the context of the escalating worldwide prevalence of obesity and type-II diabetes, the harmful consequences of NAFLD are emerging as a burgeoning confrontation within the realm of public health. NAFLD is intricately intertwined with obesity, dyslipidemia, hyperglycemia, hypertension, and liver function test abnormalities [1]. NAFLD comprises a range of disorders, varying from non-alcoholic fatty liver (NAFL), which is generally considered a less severe illness, to non-alcoholic steatohepatitis (NASH), a more critical disorder. It is noteworthy that NAFLD has the potential to progress to advanced stages, including cirrhosis and fibrosis [2,3]. An estimated 5.7–17% of Americans are thought to be afflicted by NASH [4]. Visceral adiposity is linked with an elevated probability of NAFLD, while metabolic syndrome is a recognized predisposing factor for the onset of cardiovascular disease [5]. The vagus nerve assumes a pivotal starring role in the regulation of metabolism, influencing processes such as glucose uptake, gluconeogenesis, and fatty acid metabolism. VNS has demonstrated various favorable effects on metabolism, including enhanced glucose control and heightened fatty acid oxidation. While the precise mechanisms through which VNS exerts its influence on metabolism remain incompletely understood, research has suggested its involvement in multiple pathways, with the activation of peroxisome proliferator-activated receptor (PPAR) being one proposed mechanism [6]. The vagus nerve serves as the primary innervation of viscera, establishing a crucial link between essential metabolic cues and the central nervous system (CNS). Notably, PPARγ exhibited enrichment in vagal sensory neurons. High-fat-diet feeding has been associated with a reduction in PPARγ expression. Intriguingly, the deletion of PPARγ in vagal nerve cells enhanced HFD-induced thermogenesis and facilitated the reprogramming of white adipocytes into a brown-like adipocyte cell phenotype [7].

VNS is an important afferent conduit for the perception of peripheral sensations, and it has been shown to help regulate obesity [8]. This is because it allows the brain to begin responses by activating the immunological, metabolic, endocrine, and autonomic nervous systems [9]. The vagal anti-inflammatory response is triggered by the brain’s detection of inflammation, which controls both localized and systemic inflammatory reactions [10]. Diverse research has documented the modulatory effect of VNS on amygdala activity, which suggested a role for VNS in lowering cue-driven intake [11,12]. Moreover, cervical VNS decreased hippocampal activity; this area is linked to memory and remembrance functions and is also active in response to desiring elements of foodstuff [11].

Interventions targeted at increasing vagal activity include non-invasive techniques, like VNS, and invasive procedures, like vagus nerve stimulation (VNS), which uses electrodes that are surgically inserted and connected to a generator unit [13]. VNS is a simple therapeutic modality that stimulates the vagus nerve’s auricular branch, which is located in the exterior of the ear, to provide autonomic benefits. For instance, even in young, healthy individuals, it reduces sympathetic nerve activity and increases spontaneous heart baroreflex sensitivity [14,15]. Therefore, our study was primarily aimed at assessing whether VNS could alleviate obesity-induced NAFLD. This investigation was inspired by the well-documented link between obesity, diabetes, and vagal activity alterations [16,17,18]. The objective was to explore an approach that would mitigate the need for long-term medications, many of which are associated with sustained adverse effects, particularly impacting the cardiovascular system, in the treatment of obesity-related conditions. Moreover, a significant portion of obese patients concurrently grapple with NAFLD. Hence, there remains a pressing need for secure anti-obesity interventions that not only address weight loss efficacy but also prioritize the enhancement of liver function. Our particular focus lies in the exploration of the clinical translation of neural system modulatory mechanisms as a potential foundation for therapeutic approaches to address obesity-related disorders.

## 2. Materials and Methods

### 2.1. Ethical Approval

The Ethics Committee for Animal Experimentation guidelines at the College of Medicine, Zagazig University, approved all experimental procedures (Approval number: ZU-IACUC/3/F/230/2022).

### 2.2. Animal Grouping

A total of 32 adult male albino rats (10 weeks old), each weighing between 180 and 200 g, were sourced from the animal facility located at the Faculty of Veterinary Medicine, Zagazig University. These rats were housed in steel wire cages, with a housing density of 3 to 4 rats per cage, within a controlled environment, maintaining a regulated temperature and a natural light/dark cycle. Moreover, the rats were provided unrestricted access to water throughout the study period.

Following 1 week of acclimatization to their new environment, the rats were grouped into two distinct clusters: (A) the Control group consisted of 8 rats and was fed a standard diet comprising 25.8% protein, 62.8% carbohydrates, and 11.4% fat [5]; (B) the HFD group consisted of 24 rats and was provided with a diet rich in corn oil, containing over 98% ω-6 polyunsaturated fatty acids (PUFA). This high-fat diet was composed of 21.4% fat, 17.5% protein, 50% carbohydrates, 3.5% fiber, and 4.1% ash. This obesogenic dietary regimen was maintained for 12 weeks to induce hepatic steatosis, commonly known as NAFLD [19]. The rats on HFD (=24 rats) were subdivided into three equal groups: the non-treated group (*n* = 8), the sham-stimulation group (*n* = 8), and the VNS-treated group (*n* = 8).

### 2.3. Transcutaneous Vagus Nerve Stimulation (tVNS) Intervention

As regards the tVNS-treated group, under 2% isoflurane anesthesia, tVNS (20 Hz frequency, 2 mA amplitude, 0.2 ms pulse duration) was conveyed for 30 min per day for 6 weeks after establishment of NAFLD using a digital TENS device (Model: TENS 7000TM, Sherman Oaks, CA, USA). This was achieved by utilizing two magnetically charged electrodes with opposing charges—one placed inside (cathode) and the other outside (anode)—at the auricular concha region of each ear, as outlined in previous descriptions [20]. The results of the earlier research served as the foundation for the tVNS parameters.

In the HFD sham group (20 Hz, 2 mA, 0.2 ms), under 2% isoflurane anesthesia, the electrodes were positioned on the auricular margin, which is not innervated by the vagus, based on other transcutaneous VNS investigations that used sham stimulation as a comparative group [21,22]. Throughout the acute stimulation period, when data from all rats during both baseline and endpoint sessions were averaged, it became evident that active tVNS led to a slight, yet statistically significant, decrease in heart rate (baseline: 356 ± 15 bpm vs. active tVNS: 348 ± 14 bpm; *p* ≤ 0.05). This observation suggests that the activation of vagal fibers had occurred.

### 2.4. Analytical Evaluation of Body Mass Index (BMI) and Abdominal Circumference (AC)

Total body weight in grams was assessed using a digital scale. The animals’ weights were recorded 1 day before the commencement of the experiments, and subsequently twice a week, throughout the study period. Final weight measurements were also taken on the last day of the experiment. Additionally, the nose-to-anus length in centimeters (cm) was measured after the experiments. These measurements were essential for the computation of the body mass index (BMI) by dividing the body weight (gm) by the square of the length (cm^2^) [23]. The measuring tape is wrapped around the belly slightly in front of the hind limbs for the measurement of abdominal circumference (AC) and the chest directly behind the forelimbs for the measurement of thoracic circumference (TC). Next, information was plotted in each designated rat’s record at the start and end of the study duration to calculate the AC/TC ratio [24].

### 2.5. Collection of Specimens

At the conclusion of the experiment, blood samples were collected and centrifuged, and the resulting sera were subsequently utilized to measure additional indicators of liver function. The livers were removed from the abdominal cavity, dissected, and then blotted dry with filter sheets, cleaned in ice-cold phosphate-buffered saline (PBS), and weighed to calculate the coefficient [23] before being cut into sections for additional biochemical and histological evaluations.

### 2.6. Evaluation of Insulin Resistance

Sera were obtained from retro-orbital blood samples centrifuged at 1500× *g* for 20 min following an overnight fast, and stored at −20 °C for further processing for biochemical analysis. Using a sugar detection kit (Biosource Europe S.A. Brussels, Belgium, Cat. No. EIAGLUC), the serum glucose level (mmol/L) was measured colorimetrically, as previously mentioned [25]. Rat insulin ELISA Kits (Sigma-Aldrich, Cairo, Egypt, Cat. No. EZRMI) were used to measure the serum insulin level (pmol/L) in accordance with the manufacturer’s instructions. Insulin had a standard curve range of 1.5–48 mIU/L and a sensitivity of 0.1 mIU/L. The reaction was stopped by adding an acidic solution, and absorbance measurements were taken using a multimode microplate reader (Synergy, Santa Clara, CA, USA) at 450 nm. The HOMA-IR, which stands for Homeostatic Model Assessment of Insulin Resistance, is calculated using the following formula: HOMA-IR = Fasting serum insulin (μIU/mL) × Fasting serum glucose (mmol/L)/22.5.

### 2.7. Evaluation of Blood Lipid Profile

The total cholesterol (CHOL) and triglycerides (TG) levels in the serum were measured using an enzymatic colorimetric method with specific kits (Spinreact, Girona, Spain, Cat. No. CHOD-POD and Cat. No. GPO-POD, respectively). The absorbance was measured at a wavelength of 510 nm using a spectrophotometer. The ratio of sample to reagent was kept at 1:150, as per the instructions provided by the manufacturer.

The levels of high-density lipoprotein-cholesterol (HDL-c) were determined using a similar enzymatic colorimetric method and an HDL cholesterol assay kit (Biodiagnostic, Cairo, Egypt, Cat. No. CH 12 30), following the protocols recommended by the manufacturer.

The levels of low-density lipoprotein-cholesterol (LDL-c) in the serum were calculated using the formula: LDL-c = TC − HDL − (TG/5) [26].

### 2.8. Biochemical Analysis of Liver Function

A commercially available kit was used to quantify the levels of serum alanine aminotransaminase (ALT) and aspartate aminotransaminase (AST), provided by ELITech Clinical Systems, Sées, France), albumin (AB 10 10), supplied by Biodiagnostic, Dokki, Giza, Egypt), and total bilirubin. All measurements were carried out following the guidelines provided by the manufacturer.

### 2.9. Measurement of Hepatic Triglyceride and Cholesterol Content

Following the extraction of tissue lipids, as per the approach explained by Folch et al. [27], the hepatic triglyceride (TG) and cholesterol (CHOL) levels were determined in accordance with the procedure outlined by Foster and Dunn [28]. In this process, a liver mixture composed of 25 mg of frozen tissue and 100 μL of phosphate buffer saline (PBS) with a weight/volume (*w*/*v*) ratio at a pH of 7.4 was prepared. This mixture was then combined with 500 μL of an extraction solvent comprising chloroform and methanol in a 2:1 ratio, and the resultant mixture was homogenized. Subsequently, it was centrifuged at 2500 rpm for 5 min at a temperature of 4 °C. The obtained supernatant was collected, and the mixture was further subjected to a wash with 100 μL of 0.9% normal saline (NS) at room temperature, allowing the components to separate into distinct layers. The lower lipid layer was isolated and transferred to another test tube, where it was subjected to evaporation at 70 °C using a water bath. After drying, a 10 μL aliquot of the resultant mixture was combined with 100 μL of PBS for the measurement of triglyceride (TG) and cholesterol (CHOL) content. This was achieved using conventional TG and CHOL kits (Sigma-Aldrich, Cairo, Egypt, Cat. No. MAK266, MAK043) on a biosystems bioanalyzer. The absorbance was recorded at 570 nm, and the unit of measurement was expressed as milligrams per gram (mg/g) of liver tissue.

### 2.10. Evaluation of Liver Oxidative Stress Markers

Hepatic tissues underwent processing to obtain a 10% homogenate (*w*/*v*) using a 20 mM hydroxymethyl buffer at a pH of 7.4. Following homogenization, supernatants were saved, subsequent to centrifugation of the homogenates at 1500× *g* for 30 min at a temperature of 4 °C. These supernatants were then utilized for the estimation of oxidative stress markers. The quantity of malondialdehyde (MDA), serving as an indicator of lipid peroxidation, was carried out using a biodiagnostic kit, following the method outlined by Varshey and Kale [29].

Hepatic superoxide dismutase (SOD) activity was evaluated using phenazine methosulfate (PMS) and based on the inhibition of nitro-blue tetrazolium, following the method outlined by Misra and Fridovich [30].

Furthermore, the activity of glutathione S-transferase (GST) was estimated using 1-chloro-2,4-dinitrochlorobenzene, in accordance with Rajurkar et al. [31]. The measurement of GST activity is expressed as micromoles of CDNB-GSH conjugate formed per minute per milligram of protein. The assay for glutathione peroxidase (GPx) activity in the liver extract was conducted following the protocol established by Paglia and Valentine [32]. This method relies on the reduction in peroxides at a wavelength of 340 nm, facilitated by the presence of nicotinamide adenine dinucleotide phosphate (NADPH). The activity of GPx, or Glutathione Peroxidase, is measured in units (U), where 1 unit is defined as the amount of enzyme needed to oxidize 1 nanomole (nM) of NADPH in 1 min. The kits used for these measurements, including those for MDA (Cat. No. MAK085), SOD (Cat. No. 19160), GST (Cat. No. MAK453), and GPx (Cat. No. MAK437), were obtained from Spectrum Co. (Sigma-Aldrich, Cairo, Egypt).

### 2.11. Assessment of Hepatic Inflammatory Markers

The serum TNF-α and IL-6 were measured using ELISA. Commercially available kits from ALPCO (Catalog No. 45-TNFRT-E01.1) and LsBio (Catalog No. LS-F2482) were used for this purpose. The absorbance was recorded at a wavelength of 450 nm, following the guidelines provided by the manufacturer.

### 2.12. Histopathological Evaluation of Liver Tissue

Sections of 5 µm thickness were prepared for Hematoxylin and Eosin staining (H&E) to examine the hepatic architecture, following a previously described protocol [33]. Hepatic damage was assessed using the NAFLD Activity Score (NAS) established by The Pathological Committee of the NASH Clinical Research Network [34]. The NAS scores were calculated as the sum of individual scores for steatosis (0 = <5%, 1 = 5–33%, 2 = 34–66%, 3 = >66%), lobular inflammation (0 = no foci, 1 = <2 foci per 200× field, 2 = 2–4 foci per 200× field, 3 = >4 foci per 200× field), and ballooning (0 = none, 1 = rare or few, 2 = many or prominent). A NAS score of ≥5 was defined as Non-Alcoholic Steatohepatitis (NASH), NAS scores of 2–5 were categorized as borderline NASH, and NAS scores of ≤2 indicated simple steatosis.

For the evaluation of hepatic fibrosis, sections were stained with Sirius red following the previously outlined methodology [35]. Fibrosis scoring included stage 1, characterized by perivenular and perisinusoidal fibrosis (further subdivided into 1a and 1b based on the extent of the deposit), as well as isolated periportal fibrosis (1c); stage 2 encompassed portal and central fibrosis without bridging fibrosis; stage 3 represented bridging fibrosis; and stage 4 indicated cirrhosis [34].

### 2.13. Immunohistochemical Analysis of Hepatic Tissue

Caspase-3 and tumor necrosis factor-alpha (TNF-α) were employed as markers for apoptosis and inflammation, respectively. Following the previously described protocol [35], hepatic sections were rehydrated using a series of ethanol solutions and then washed with distilled water. Heat-mediated antigen retrieval was carried out by immersing the sections in 10 mM citrate buffer (pH 6.0, epitope retrieval solution) and heating them in a water bath at 98 °C for 20 min. The sections were allowed to cool for 20 min and then washed under running tap water. Subsequently, endogenous peroxidase activity was blocked by treating the sections with 0.3% hydrogen peroxide for 10 min at room temperature (RT). After rinsing in PBS (Phosphate-buffered saline), non-specific binding was prevented through a 1-h incubation in 5% normal goat serum at RT. Following this blocking step, the sections were incubated with the primary antibodies for caspase-3 (Novus, rabbit polyclonal IgG, Cat. #NB100-56113 at a dilution of 1/200) and TNF-α (Abcam, rabbit polyclonal antibody, ab6671 at a dilution of 1/100) overnight at RT.

Next, the sections were incubated with a secondary biotinylated antibody for 20 min following a PBS rinse. An enzyme conjugate, “Streptavidin-Horseradish peroxidase” solution, was then applied to the sections for 10 min. Secondary antibody binding was visualized using 3,3-diaminobenzoic acid (DAB) as a chromogen. Before each of the aforementioned steps, the sections were washed with PBS. Finally, stained slides were examined using light microscopy (The Leica DM500, Leica Microsystems Co., Wetzlar, Germany) with Leica ICC50 W Camera Module).

### 2.14. Morphometrical Analysis

The image analysis was carried out using the “Image J” image processing software (Version 1.50, dated 23 April 2019). To quantify the immunoexpression of TNF-α and the areas positively labeled for caspase-3 (depicted as brown-stained areas), the analysis was performed at a magnification of ×400. The quantification of the areas marked in red from Sirius red staining was conducted at a magnification of ×100. For each specimen in the different groups, five non-overlapping and randomly selected fields from the respective slide were evaluated.

### 2.15. Transmission Electron Microscopic Study (TEM)

The liver samples (1 mm^3^ thick) were post-fixed in 1% osmium tetra-oxide, dried, and preserved in resin after being preserved for 24 h in a solution of 2.5% glutaraldehyde and 2.5% paraformaldehyde in phosphate buffer. Thin sections were then placed on copper grids to be dyed with uranyl acetate and lead citrate. The sections were examined using a JEM-2100 transmission electron microscope (TEM) in order to identify ultrastructural changes.

### 2.16. Statistical Analysis

Quantitative data will be compared using ANOVA, and then post-hoc analysis (Tukey test). The data are presented in the format of mean ± standard error (S.E.). *p*-values will be regarded as statistically significant if they are less than 0.05. Initially, normality plots and the Shapiro-Wilk test were used to carefully evaluate the data for normality.

## 3. Results

### 3.1. VNS Improved Weight Gain, Liver Weight, and Hepatic Coefficient Induced by HFD

**Figure 1:**
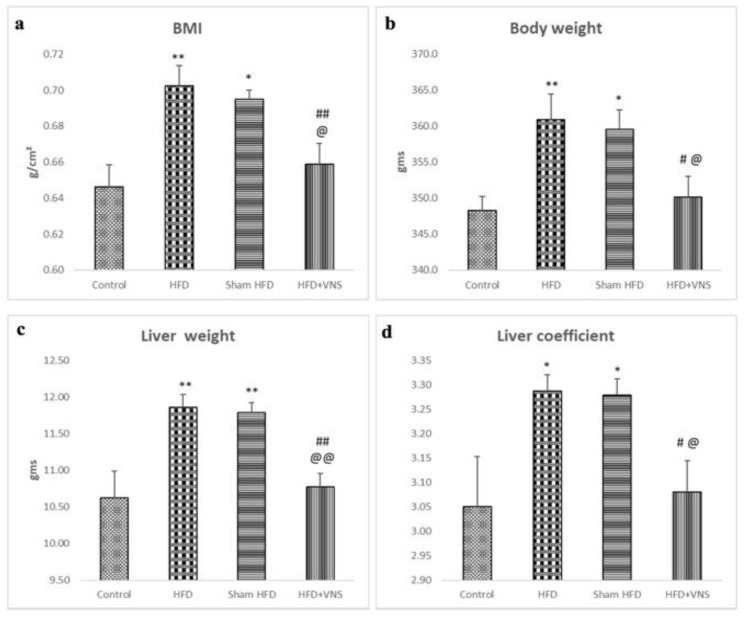
Modulatory impact of tVNS on Body weight, Liver weight, and Liver coefficient: (**a**) Body Mass Index (BMI); (**b**) Body Weight; (**c**) Liver Weight; and (**d**) hepatic Coefficient. Data were presented as mean ± SEM and analyzed using ANOVA. Tukey’s post hoc test was used to compare means between groups. Statistical significance was set at *p* < 0.05. * *p* < 0.05 related to the normal control group; ** *p* < 0.001 related to the normal control group; # *p* < 0.05 related to the corresponding values of the HFD group; ## *p* < 0.001 related to the HFD group; @ *p* < 0.05 related to the corresponding values of the Sham HFD group and @@ *p* < 0.001 related to the corresponding values of the Sham HFD group.

At the launching of the experiment, there were no significant differences in body weight among the groups. However, at the end of the study, the rats in the HFD groups exhibited a noticeable increase in body weight, accompanied by a significant rise in BMI, AC, and AC/TC ratio when compared to the control group. Conversely, the rats in the HFD+VNS group demonstrated a significant decrease in all obesity-related indices when compared to the HFD group. There were no significant differences observed between the HFD Sham group and the HFD group (Figure 1a,b). The rats in the HFD and HFD-Sham groups displayed significantly higher liver weight and liver coefficient compared with the control group. Vagus Nerve Stimulation mitigated the increase in liver weight induced by the HFD, resulting in significantly lower liver weight and liver coefficient in the rats of the HFD+VNS group compared with the HFD and sham groups (Figure 1c,d).

### 3.2. VNS Reduced HFD-Induced Accumulation of Hepatic Triglyceride and Cholesterol

**Figure 2:**
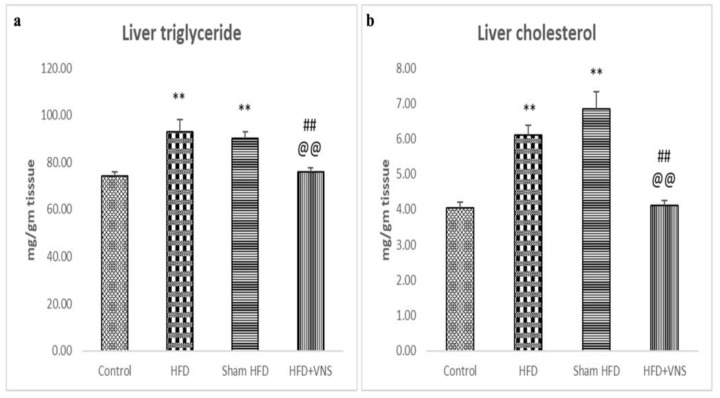
Modulatory effect of tVNS on the Hepatic Triglyceride and Cholesterol content: (**a**) Liver Triglyceride; (**b**) Liver Cholesterol. Data were presented as mean ± SEM and analyzed using ANOVA. Tukey’s post hoc test was used to compare means between groups. Statistical significance was set at *p* < 0.05. *p* < 0.05 related to the normal control group; ** *p* < 0.001 related to the normal control group; *p* < 0.05 related to the corresponding values of the HFD group; ## *p* < 0.001 related to the HFD group; *p* < 0.05 related to the corresponding values of the Sham HFD group and @@ *p* < 0.001 related to the corresponding values of the Sham HFD group.

Compared to the control rats on a regular diet, the HFD-fed group’s hepatic tissue showed a significant accumulation of cholesterol and triglycerides. However, in contrast to the HFD and sham HFD groups, VNS dramatically decreased hepatic triglyceride and cholesterol deposition in the HFD+VNS group, as demonstrated in Figure 2.

### 3.3. VNS Mitigated HFD-Induced Alterations in Lipid Profiles and Hepatic Functions

**Figure 3:**
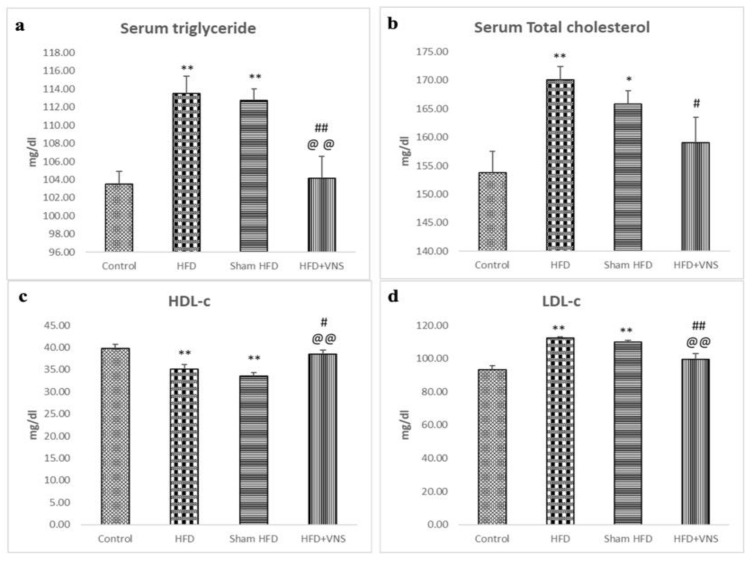
Modulatory effect of tVNS on the Lipid Profile: (**a**) Serum Triglyceride; (**b**) Serum Total Cholesterol; (**c**) HDL-c; and (**d**) LDL-c. Data were presented as mean ± SEM and analyzed using ANOVA. Tukey’s post hoc test was used to compare means between groups. Statistical significance was set at *p* < 0.05. * *p* < 0.05 related to the normal control group; ** *p* < 0.001 related to the normal control group; # *p* < 0.05 related to the corresponding values of the HFD group; ## *p* < 0.001 related to the HFD group; *p* < 0.05 related to the corresponding values of the Sham HFD group and @@ *p* < 0.001 related to the corresponding values of the Sham HFD group.

The investigations showed that in comparison to the control group, the serum TG, CHOL, and LDL-c levels of the HFD-fed rats were considerably raised, with a significant drop in the serum levels of HDL-c. However, the rats on HFD that received VNS showed dramatically reduced serum TG, CHOL, and LDL-c, and elevated HDL-c levels compared to the HFD and sham HFD groups (Figure 3).

**Figure 4:**
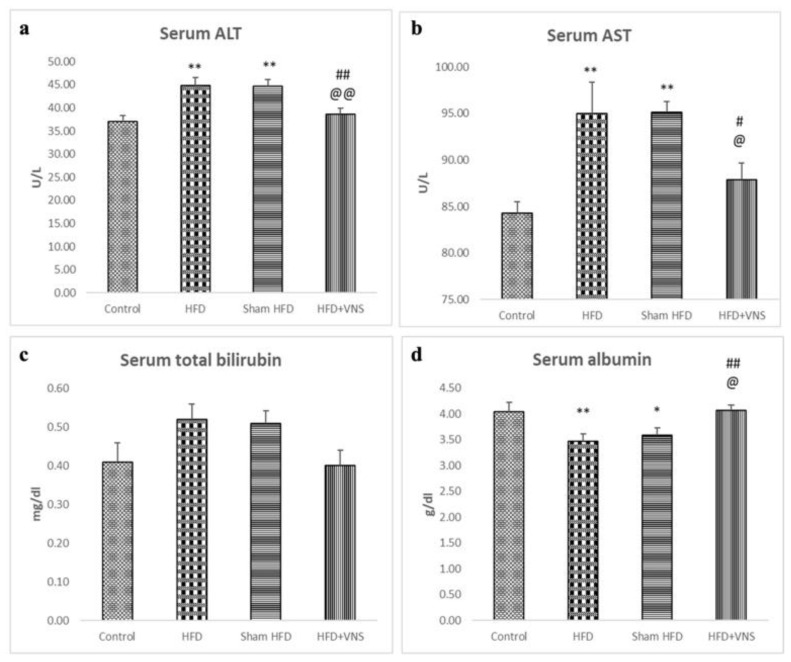
Modulatory effect of tVNS on Liver Function: (**a**) Serum ALT; (**b**) Serum AST; (**c**) Serum Total Bilirubin; and (**d**) Serum Albumin. Data were presented as mean ± SEM and analyzed using ANOVA. Tukey’s post hoc test was used to compare means between groups. Statistical significance was set at *p* < 0.05., * *p* < 0.05 related to the normal control group; ** *p* < 0.001 related to the normal control group; # *p* < 0.05 related to the corresponding values of the HFD group; ## *p* < 0.001 related to the HFD group; @ *p* < 0.05 related to the corresponding values of the Sham HFD group and @@ *p* < 0.001 related to the corresponding values of the Sham HFD group.

Furthermore, the serum albumin levels decreased, whereas the ALT, AST, and serum total bilirubin levels significantly increased, in the HFD and sham HFD groups when compared to the control group. In contrast, the HFD+VNS group exhibited notably elevated albumin levels and significantly reduced levels of ALT, AST, and serum total bilirubin (Figure 4).

### 3.4. VNS Alleviated the Hepatic Inflammatory Indicators Induced by HFD

**Figure 5:**
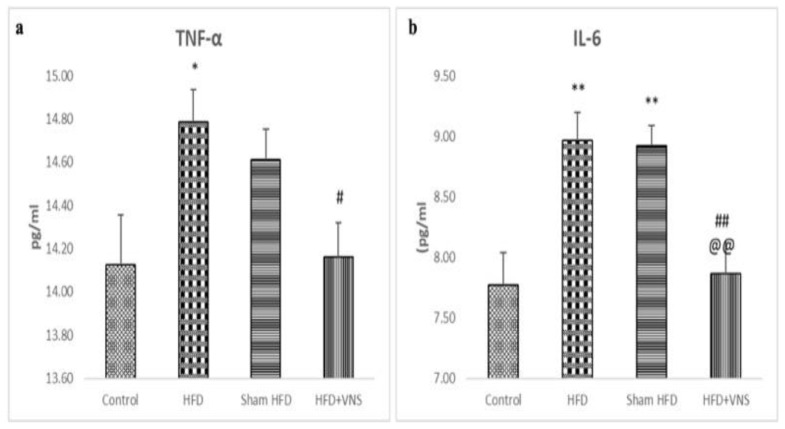
Modulatory effect of tVNS on the Hepatic Inflammatory markers: (**a**) TNF-α; (**b**) IL-6. Data were presented as mean ± SEM and analyzed using ANOVA. Tukey’s post hoc test was used to compare means between groups. Statistical significance was set at * *p* < 0.05. ** *p* < 0.001 related to the normal control group; # *p* < 0.05 related to the corresponding values of the HFD group; ## *p* < 0.001 related to the HFD group; *p* < 0.05 related to the corresponding values of the Sham HFD group and @@ *p* < 0.001 related to the corresponding values of the Sham HFD group.

The TNF- and IL-6 levels in the hepatic tissue of the HFD+VNS group were significantly lower than those of the HFD and sham HFD groups, indicating a marked reduction in the HFD-induced hepatic inflammatory process. Nonetheless, the hepatic tissue from the HFD and sham HFD groups showed significantly higher levels of TNF-α and IL-6 than that of the control group (Figure 5).

### 3.5. VNS Improved the Disrupted Hepatic Oxidative Stress Induced by HFD

**Figure 6:**
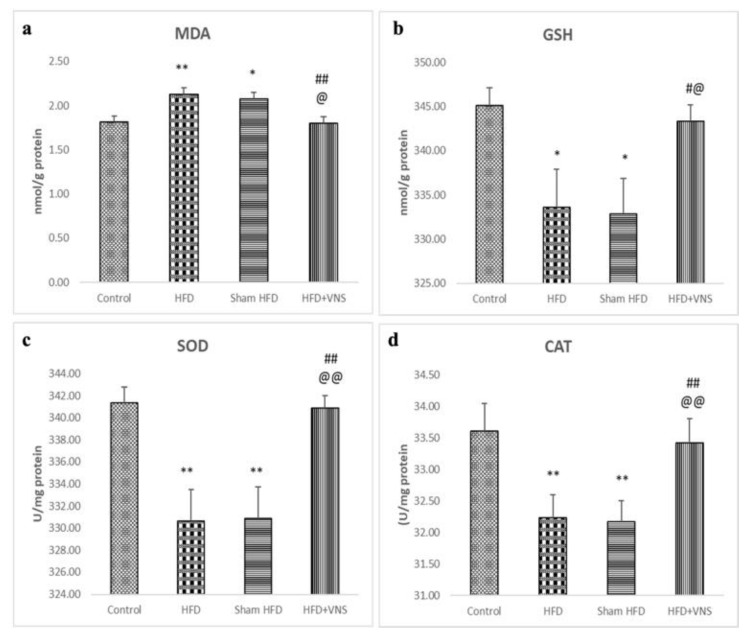
Modulatory effect of tVNS on Hepatic oxidative stress markers: (**a**) MDA; (**b**) GSH; (**c**) SOD; and (**d**) CAT. Data were presented as mean ± SEM and analyzed using ANOVA. Tukey’s post hoc test was used to compare means between groups. Statistical significance was set at *p* < 0.05. * *p* < 0.05 related to the normal control group; ** *p* < 0.001 related to the normal control group; # *p* < 0.05 related to the corresponding values of the HFD group; ## *p* < 0.001 related to the HFD group; @ *p* < 0.05 related to the corresponding values of the Sham HFD group and @@ *p* < 0.001 related to the corresponding values of the Sham HFD group.

Compared to the control group, both the HFD and sham HFD groups exhibited a significant rise in hepatic MDA levels, along with significantly reduced activities of GST, SOD, and CAT. Conversely, in the HFD+VNS group, VNS effectively ameliorated these alterations (Figure 6).

### 3.6. VNS Mitigated Histological Changes Induced by HFD in Hepatic Tissue

#### 3.6.1. H & E

**Figure 7:**
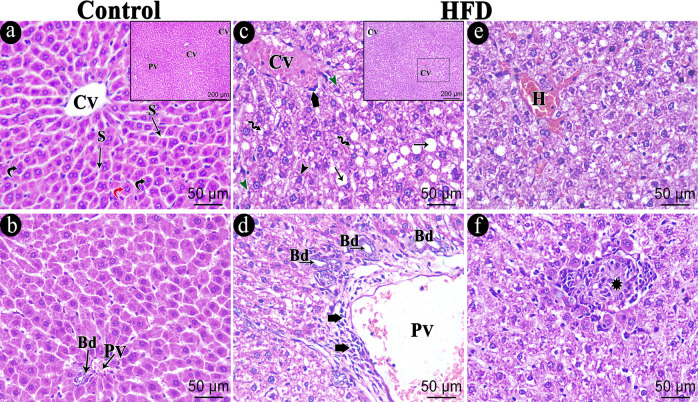
Photomicrograph of the H&E-stained hepatic sections of the studied groups. (**a**,**b**) Hepatic sections of the control group showing part of a hepatic lobule with central vein (CV) and tightly packed cords of hepatocytes with arrow radiating blood sinusoids (S) in between. Hepatocytes exhibit rounded vesicular nuclei and acidophilic cytoplasm (Red curved arrow) with numerous binucleated hepatocytes (black curved arrow). The portal area contains a portal vein (PV) and bile duct (Bd). (**c**–**f**) HFD group showing central vein (CV) surrounded by swollen hepatocytes with variable cytoplasmic vacuolations, either foamy cytoplasm (Black Arrowhead), multiple defined small vacuoles (Zigzag arrow) or large coalesced vacuoles (Arrow). Some hepatocytes have shrunken, dark-stained nuclei (Green Arrowhead). The portal area shows dilated, congested portal vein (PV) and bile duct proliferation (Bd). Notice the mononuclear cellular infiltration (Short thick arrow) within the portal area and around the central vein. Hemorrhage (H) in addition to necrotic foci (star) are also seen within the hepatic sections. The necrotic focus is formed of a collection of cell debris and infiltration of lymphocytic cells. Scale bar = 50 µm and inset Scale bar = 200 µm.

The control group’s hepatic sections revealed closely spaced hepatocyte cords emanating from the central vein, with thin blood sinusoids separating them. Numerous binucleated hepatocytes were seen, along with rounded vesicular nuclei and acidophilic cytoplasm (Figure 7a). The hepatic artery, bile duct, and portal vein were all located in the portal tract (Figure 7b). The hepatic tissue depicted swollen hepatocytes with varying cytoplasmic vacuolations, either several defined tiny vacuoles or large consolidated vacuoles with ballooning degeneration (Figure 7c), the HFD generated non-alcoholic steatohepatitis with substantial micro and macro steatosis. The bile duct proliferated and the portal region displayed dilated, congested portal veins (Figure 7d). The portal region and the area surrounding the central vein (Figure 7c,d) both showed mononuclear cellular infiltrations (NASH score ranging between 5 and 6). Within the hepatic sections, necrotic foci and hemorrhage were also observed (Figure 7e,f).

**Figure 8:**
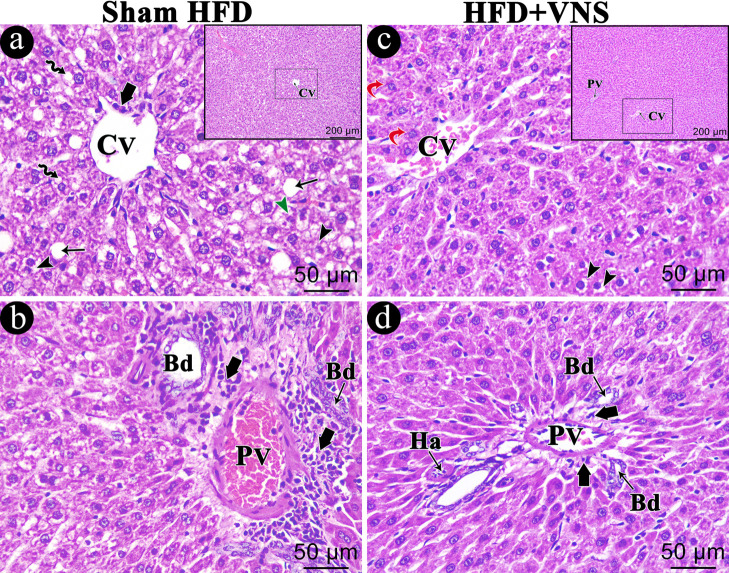
Photomicrograph of the H&E-stained hepatic sections of the studied groups. (**a**,**b**) Sham HFD group shows the exact similar histopathological changes within the HFD group showing swollen hepatocytes with variable cytoplasmic vacuolations either foamy cytoplasm (Black Arrowhead), multiple defined small vacuoles (Zigzag arrow) or large coalesced vacuoles (arrow). hepatocytes with shrunken dark stained nuclei (Green Arrowhead) are also seen. The portal area shows dilated congested portal vein (PV) and bile duct proliferation (Bd). Mononuclear cellular infiltrations (Short thick arrow) appear within the portal area and around the central vein. Within the HFD+VNS group (**c**,**d**), most hepatocytes appear normal (Red curved arrow), and few cells contain some cytoplasmic vacuoles (black Arrowhead). In addition, mild cellular infiltrations (Short thick arrow) around components of a portal tract (portal vein (PV), Hepatic artery (Ha), and bile duct (Bd)). Scale bar = 50 µm and inset Scale bar = 200 µm.

The HFD Sham group exhibited analogous histopathological changes to those observed in the HFD group (Figure 8a,b). The hepatic lesions significantly improved with vagal stimulation, which partially restored the liver’s normal architecture, with the majority of hepatocytes appearing normal and a small number of cells containing some cytoplasmic vacuoles (Figure 8c). Furthermore, there were slight cellular infiltrations in the portal area (Figure 8d).

#### 3.6.2. Sirius Red Staining

**Figure 9:**
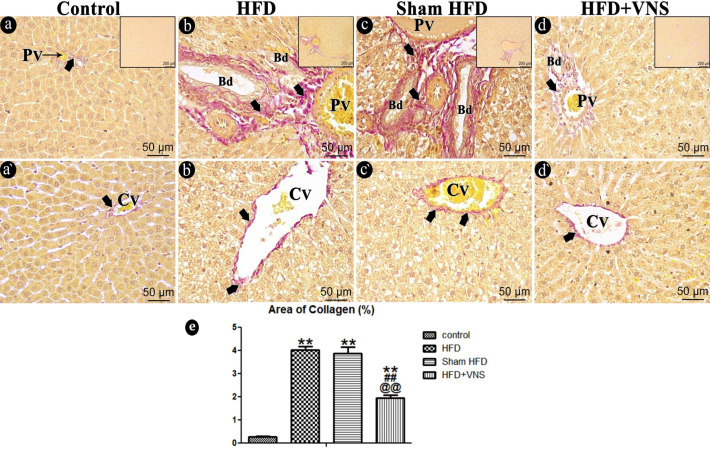
Photomicrograph of the studied groups’ Sirius red-stained hepatic sections. The control group (**a**,**a’**) displays only a small amount of collagen fibers (arrow) encircling portal (PV) and central (CV) veins. HFD (**b**,**b’**) and the Sham HFD (**c**,**c’**) groups show dense collagen fiber deposition (arrow) around the central vein (Cv) and within the portal area around the portal vein (PV) and bile ducts (Bd) duct. While the HFD+VNS group (**d**,**d’**) shows a moderate amount of collagen fibers (arrow) surrounding the portal vein (PV) and bile ducts (Bd) duct and around the central vein as well. (**e**) % area of Sirius red staining in hepatic specimens. Data were expressed as Mean ± SEM by ANOVA, followed by Tukey post hoc test, ** *p* < 0.001 related to the normal control group; ## *p* < 0.001 related to the HFD group; and @@ *p* < 0.001 related to the corresponding values of the Sham HFD group. Scale bar = 50 µm and inset Scale bar = 200 µm.

The liver sections from the control group, assessed through Sirius red staining and graded as 0, exhibited no indications of fibrosis, except for a minimal deposition of collagen fibers surrounding the central vein and in the portal region (Figure 9a,a’).

In contrast, the HFD group demonstrated extensive collagen fiber deposition within the portal tract, extending into a few lobular septa, and around the central vein, with scores of 2–3 (Figure 9b,b’). These findings were consistent with those observed in the sham HFD group (Figure 9c,c’). Collagen fiber deposition around the central vein and inside the portal locations was significantly reduced in the vagal-stimulated HFD group (scoring 1c–2) (Figure 9d,d’).

Statistical analysis of the percentage of Sirius red-positive stained areas within the HFD group showed a highly significant increase (*p* < 0.001) compared to the control group. Conversely, vagal stimulation led to a highly significant reduction (*p* < 0.001) in the area occupied by collagen fibers when compared to the HFD group (Figure 9e).

#### 3.6.3. Immunostaining

**Figure 10:**
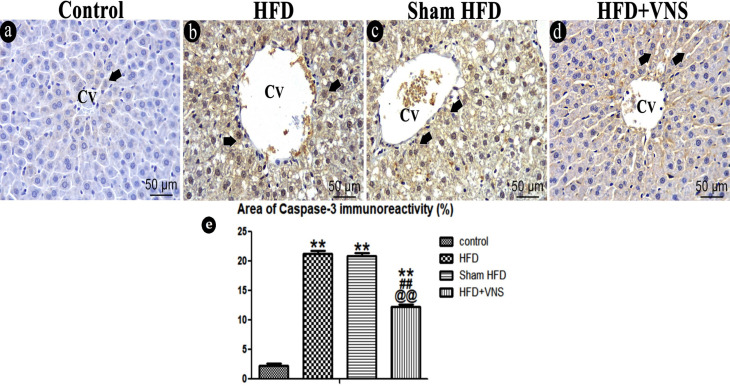
Photomicrograph of the apoptotic marker caspase-3 in the hepatic sections of various studied groups. (**a**) The control group shows a very weak positive cytoplasmic caspase-3 reaction in some hepatocytes (Short thick arrow). Hepatocytes of the HFD (**b**) and Sham HFD (**c**) groups similarly exhibit a marked increase in caspase-3 immunoreactivity (Short thick arrow). (**d**) HFD+VNS group shows moderate caspase-3 immunoreactivity (Short thick arrow) within some hepatocytes. Note: the central vein (CV) presence in hepatic sections. (**e**) % area of caspase-3 immunostaining in hepatic specimens. Data were expressed as Mean ± SEM by ANOVA, followed by Tukey post hoc test, ** *p* < 0.001 related to the normal control group; ## *p* < 0.001 related to the HFD group; and @@ *p* < 0.001 related to the corresponding values of the Sham HFD group. Scale bar = 50 µm.

In the Caspase-3 immunostained hepatic sections of the control group, only a few hepatocytes exhibited a very mild positive cytoplasmic reaction. Administration of the HFD led to a significant increase (*p* < 0.001) in caspase-3 immunoreactivity when compared to the control group. This pattern remained consistent within the sham HFD group. However, following vagal stimulation, there was a significant reduction (*p* < 0.001) in caspase-3 immunoreactivity when compared to the HFD group (Figure 10).

**Figure 11:**
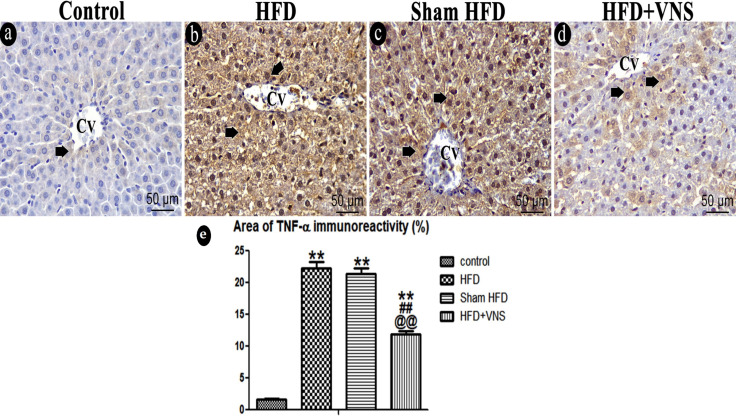
Photomicrograph of the inflammatory marker TNF-α within the studied groups. (**a**) very weak immunoreaction for TNF-α protein expression in the cytoplasm of some hepatocytes of the control group (short thick arrow). (**b**) Hepatocytes of the HFD group show Strong positive immunoreaction for TNF-α (short thick arrow). (**c**) Sham HFD group similarly shows a marked increase in TNF-α positive immunoreaction. (**d**) HFD+VNS group shows positive immunoreaction for TNF-α in the cytoplasm of some hepatocytes (short thick arrow). Note: the central vein (CV) presence in hepatic sections. (**e**) % area of TNF-α immunostaining in hepatic specimens. Data were expressed as Mean ± SEM by ANOVA, followed by Tukey post hoc test, ** *p* < 0.001 related to the normal control group; ## *p* < 0.001 related to the HFD group; and @@ *p* < 0.001 related to the corresponding values of the Sham HFD group. Scale bar = 50 µm.

A weak positive cytoplasmic response was observed in a small number of hepatocytes in the control group in the hepatic sections stained for TNF-α. After consuming the HFD, TNF-α immunoreactivity was significantly (*p* < 0.001) elevated compared to the control group. When compared to the HFD group, however, the Sham HFD group showed no discernible difference. Vagal stimulation resulted in a noteworthy and statistically significant decrease (*p* < 0.001) in TNF-α immunoreactivity compared to the Sham HFD group (Figure 11).

### 3.7. VNS Mitigated Ultrastructural Changes Induced by HFD in Hepatic Tissue

**Figure 12:**
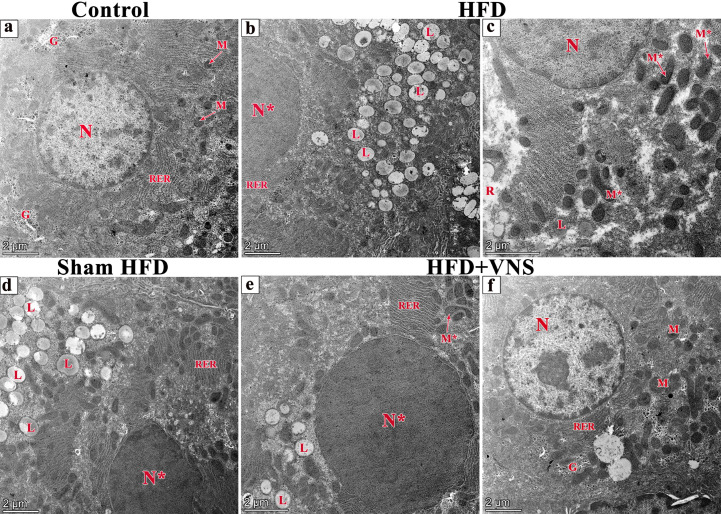
Electron micrographs of ultrathin sections of studied groups (**a**) the liver of the control group showing polyhedral-shaped hepatocyte-containing large, euchromatic rounded nucleus (N) with chromatin masses surrounded by a nuclear membrane. The cytoplasm contains abundant mitochondria (M), rough endoplasmic reticulum (RER), and glycogen granules (G). (**b**,**c**) ultrathin sections of the HFD group show numerous lipid droplets with variable size and shape (L) within the cytoplasm of the hepatocytes and rarefaction of cytoplasm (R). A heterochromatic nucleus (N*) and some irregular-shaped mitochondria (M*) are also seen. (**d**) The ultrathin section of the Sham HFD group has the same changes within the HFD group. However, ultrathin sections of the HFD+VNS group; (**e**) show few lipid droplets (L), irregular-shaped mitochondria (M*), and heterochromatic nucleus (N*) in addition to the others (**f**) showing preserved ultrastructural picture including mitochondria (M) and rough endoplasmic reticulum (R), and glycogen granules (G). Scale bar = 2 µm.

Examination of the ultrathin hepatic sections in the control group revealed polyhedral-shaped hepatocytes characterized by large, rounded euchromatic nuclei with chromatin masses enclosed by an intact nuclear membrane. The cytoplasm displayed an abundance of mitochondria, rough endoplasmic reticulum, and glycogen granules (Figure 12a).

Conversely, in the HFD group, hepatocytes exhibited cytoplasmic rarefaction, numerous lipid droplets of varying size and shape, a depletion of glycogen inclusions, and irregular mitochondria. Additionally, heterochromatic nuclei with irregular membrane structures were observed (Figure 12b,c).

The hepatic sections of the Sham HFD group displayed identical changes to those seen in the HFD group (Figure 12d); however, in the sections of the HFD+VNS group, only a few lipid droplets were observed within the hepatocyte cytoplasm, along with irregular mitochondria and heterochromatic nuclei, with a preserved ultrastructure in the majority of them (Figure 12e,f).

## 4. Discussion

Obesity can cause various hepatic lesions, including NAFLD, which is a significant one. The pandemic level of obesity and fatty liver diseases has led to a dramatic expansion in the prevalence of type 2 diabetes, and other potentially fatal conditions [36]. It is well-known that NAFLD arises from a disruption in liver fat metabolism. People with NAFLD have elevated levels of the protein CD36, which helps FAs enter liver cells. Additionally, higher levels of the fat-binding proteins FABP-4 and FABP-5 in the liver are associated with increased intrahepatic TG levels. De novo lipogenesis contributes significantly to hepatic fat retention. This pathway has a crucial role in the progress of NAFLD [37]. In this study, we established an HFD model to estimate the potential moderating effect of tVNS on the metabolic syndrome associated with NAFLD, as well as the hepatic structural and functional characteristics. To investigate the mitigating effect of bilateral auricular VNS, we also measured oxidative stress and inflammatory markers to explore the potential pathways. A well-established association exists between altered vagal stimulation and obesity [24,38,39], and our observations showed a significant decline in body weight, BMI, AC, liver weight, and hepatic coefficient in the rats of HFD+VNS compared to HFD and sham HFD, which supports this association. Collectively, our findings provide insights into how vagus stimulation influences metabolic status. Of note, it was suggested that vagal sensory neurons contain a high PPARγ content and the decline in PPARγ levels in nodose ganglion cells could potentially act as a defense mechanism to prevent weight gain induced by diet [7].

Furthermore, when compared to the rats in the HFD and sham HFD groups, VNS significantly improved the lipid profile, with a significant reduction in the TG, CHOL, and LDL-c levels and an elevation in the HDL-c level. This was also accompanied by a significant increase in insulin sensitivity in the HFD+VNS rats, improving the metabolic syndrome associated with obesity, which has previously been documented [39]. Insulin resistance is crucial for the occurrence and progression of non-alcoholic fatty liver disease [40] and is closely correlated with the inflammatory process induced by obesity [41] that was improved by VNS. It is noteworthy that inflammation associated with obesity frequently stems from the expansion of abdominal white adipose tissue, which is marked by neutrophil and macrophage infiltration. This phenomenon is closely linked to decreased levels of anti-inflammatory markers like IL-10 and adiponectin and elevated levels of pro-inflammatory markers including TNFα, IL-1β, IL-6, leptin, and resistin [17,18]. Also, obesity is associated with autonomic dysfunction, manifested as increased sympathetic activation and depressed vagal tone [16]. This observation is in line with our findings, which revealed a significant increase in TNFα and IL-6 levels within the hepatic tissue of the HFD and Sham HFD groups. However, a significant reduction in these inflammatory markers was observed in the HFD+VNS group. This reduction can be attributed to the systemic anti-inflammatory impact of VNS, achieved through the restoration of the autonomic balance and “cholinergic anti-inflammatory pathway”, the motor arm of the inflammatory reflex leading to lower systemic levels of TNFα and increased levels of adiponectin [39]. In addition, VNS exerts a direct influence on the modulation of inflammation in the liver, which aligns with previous reports of reduced cardiac fibrosis and improved cardiac function in cases of chronic myocardial infarction [42], as well as the reduction in ischemia-induced inflammation and mitigation of brain injury associated with VNS [21]. Moreover, the anti-inflammatory characteristics of VNS have been the focus of recent studies, shedding light on its potential as a bioelectronic strategy for managing obesity [36].

Obesity’s pro-inflammatory state triggers oxidative stress, which is characterized by the production of reactive oxygen species (ROS) by the mitochondria that damage the hepatic environment and impair liver function [43]. Furthermore, Oxidative stress, caused by an overproduction of ROS, leads to beta-cell dysfunction and apoptosis, hampering insulin production and secretion, and exacerbates insulin resistance, hindering insulin’s ability to promote glucose uptake by cells [44,45]. Vijgen et al. (2013) observed a correlation between the rise in energy expenditure and the decline in brown adipose tissue (BAT) activity, suggesting that VNS could potentially augment energy expenditure [46]. Furthermore, the prevention of weight gain has been previously documented, involving the increase in brown adipose tissue (BAT) weight, activation of β-adrenoreceptors, elevated uncoupling protein 1 mRNA expression in BAT, and enhanced BAT-induced thermogenesis. These outcomes were achieved through the application of transcutaneous auricular vagus nerve stimulation (taVNS) in obese rats, which targeted the auricular branch of the vagus nerve [24].

Imaging studies conducted on patients undergoing cervical vagus nerve stimulation (VNS) for depression or epilepsy, as reported by Chae et al. (2003), revealed distinct activation patterns associated with acute and chronic stimulation, suggesting that the brain undergoes gradual adjustments in response to VNS [47]. This noteworthy finding raised the possibility that extended VNS treatment may enhance its effectiveness in addressing obesity, but a sufficient duration, lasting between 4 and 8 weeks, might be needed to induce the brain changes necessary for weight loss. In another study involving obese minipigs subjected to bilateral subdiaphragmatic VNS, weight gain was observed for the initial 5 weeks following stimulation, followed by stabilization for the subsequent 9 weeks of the trial [48]. This confirms that long-term stimulation can be beneficial for weight control and is in agreement with our findings concerning the modulatory effect of tVNS on obesity parameters.

Significantly, in rats with NAFLD, our experimental results have shown the mitigating effect of tVNS on the oxidative stress and inflammatory burden associated with obesity. We have noted that tVNS significantly improved the antioxidant indicators in the liver, including GPx and SOD activities, with a reduction in lipid peroxidation and MDA. The antioxidant characteristics of VNS on cardiomyocytes have previously been documented, as demonstrated by its capacity to improve sarcomere organization and improve energy metabolism. This optimization contributes to the improvement of heart function in the context of an infarcted heart. These beneficial effects have been linked to the activation of the P13K/AKT-FoxO3A-VEGF-A/B signaling cascade [49]. The beneficial impacts of vagal stimulation on the inflammatory and oxidative states associated with HFD were evident in both the liver’s architecture and its functionality. This was observed through histopathological analysis, which revealed a significant alleviation in hepatocellular damage, inflammatory cell infiltration, and fibrosis, in addition to the improvement of liver function indicators in the HFD+VNS group, which met our expectations.

Both human and animal studies have presented evidence suggesting that higher frequencies in VNS may yield more favorable outcomes. Investigations in rats, as documented by Gil et al. (2013), have indicated frequency-dependent enhancements, although no animal study has explored frequencies exceeding 30 Hz [50]. The absence of Wallerian degeneration in minipigs at 30 Hz suggests the feasibility of testing higher frequencies. It is notable that while fiber damage can be progressively mitigated with stimulation at 50 Hz through intermittent signaling or reducing the current to below 400 μA, continuous nerve stimulation at this frequency may lead to the scattered degeneration of large myelinated fibers [51]. Consequently, these findings underscore the importance of considering stimulation patterns in terms of current, frequency, duration, and on/off timing.

## 5. Limitations of the Study

Our study has limitations. Our study was carried out under isoflurane anesthesia. Although the use of isoflurane is more appropriate for long-term anesthesia, it has anti-inflammatory effects that may interfere with the anti-inflammatory effects of tVNS in the rat model. However, Isoflurane has been linked to very rare instances of acute liver injury, with a lower potential for hepatotoxicity. Our results showed that Isoflurane did not interfere with the VNS at the hepatic level, and its long-term exposure is safe. For future studies, it is suggested to add more experimental subgroups for the study of the influence of Isoflurane administration (30 min. daily for 6 weeks) on the architecture and functions of the liver in HFD-fed and standard diet-fed rats with and without VNS application. Moreover, we can compare other anesthetic medications to select the most appropriate one in the VNS rat model. These data will have to be independently confirmed, preferably in more than one model, sex, and species, and incorporating longer-term outcomes. However, given the easy implementation, safety, and tolerability profile of non-invasive VNS, there is an opportunity for the rapid transformation of these findings into clinical practice. Chronic daily tVNS can be used prophylactically to prevent NAFLD progression in obese patients. The management of such patients is currently limited to follow-up neuroimaging only. Of course, more efforts are required to determine the optimal therapeutic patterns for these applications.

## 6. Conclusions

VNS ameliorated HFD-induced NAFLD through its anti-inflammatory and anti-oxidative attributes, which led to the restoration of the hepatic architecture, improvement of hepatic function, and the modulation of the anthropometric and metabolic manifestations associated linked to obesity. These results suggest that VNS may offer a promising therapeutic approach for managing NAFLD and its associated metabolic complications. Further research is indispensable to thoroughly elucidate the mechanisms underlying the beneficial effects of VNS on NAFLD and its associated metabolic disorders.

## Data Availability

The data presented in this study are available on request from the corresponding author.
